# Horner syndrome as a complication of ultrasound-guided ablation therapy for thyroid nodules: a scoping review

**DOI:** 10.3389/fendo.2025.1607214

**Published:** 2025-06-04

**Authors:** Tianhao Xie, Yan Fu, Xiaoshi Jin, Xiangxiang Ren, Jing Zhang, Qian Sun

**Affiliations:** ^1^ Department of General Surgery, Affiliated Hospital of Hebei University, Baoding, Hebei, China; ^2^ Basic Research Key Laboratory of General Surgery for Digital Medicine, Affiliated Hospital of Hebei University, Baoding, Hebei, China; ^3^ Department of Ophthalmology, Baoding No.1 Central Hospital, Baoding, Hebei, China

**Keywords:** thyroid, Horner syndrome (HS), ablation therapy, complication, scoping review

## Abstract

**Objective:**

To synthesize evidence on Horner syndrome (HS) as a complication of ultrasound-guided ablation therapy for thyroid nodules, including its incidence, mechanisms, risk factors, and prevention strategies, to enhance ablation safety and guide future research.

**Data sources:**

Web of Science, PubMed, Cochrane Library, and Embase.

**Review methods:**

Based on the framework of the PRISMA-ScR, a search was conducted in databases up to December 31, 2024.

**Results:**

Twelve articles were included, covering Microwave Ablation (MWA), Radiofrequency Ablation (RFA), High-Intensity Focused Ultrasound (HIFU), and Percutaneous Ethanol Injection (PEI). HS incidence rates varied: MWA 0.4%-4.2%, RFA 0.1%-1.5%, HIFU 1.5%-6.7%, with PEI incidence unspecified due to insufficient data. HS mechanisms included thermal injury to the cervical sympathetic chain, nerve damage from ethanol extravasation, and mechanical compression. Risk factors included ablation zones adjacent to the middle cervical ganglion (MCG), improper ablation parameter settings (such as excessively high power or prolonged duration), and nodule locations near the inferior thyroid artery. Prevention strategies emphasized precise preoperative ultrasound localization of the CSC and MCG, optimization of the isolation belt technique, timely adjustment of ablation parameters, real-time monitoring of symptoms, and avoiding the ablation probe tip from extending beyond the nodule edge.

**Conclusion:**

HS is a rare but serious complication with varying incidence rates by technique. Risk can be reduced through precise assessment, meticulous techniques, and technological innovations. Future prospective studies are needed to clarify incidence rates, long-term prognosis, and refine clinical practice guidelines.

## 1 Introduction

Ultrasound-guided thyroid ablation therapy is a non-surgical treatment modality that achieves *in situ* elimination or volume reduction of thyroid nodules and metastatic lymph nodes by inducing coagulative necrosis through physical or chemical methods, followed by subsequent absorption by the body (1). The primary techniques include thermal ablation modalities such as Microwave Ablation (MWA), Radiofrequency Ablation (RFA), High-intensity Focused Ultrasound (HIFU), and Laser Ablation (LA), as well as chemical ablation via Percutaneous Ethanol Injection/Percutaneous Lauromacrogol Injection (PEI/PLI) (2). This approach offers multiple clinical advantages, including scar-free intervention, high precision, short procedural duration, confirmed therapeutic efficacy, minimal complications, and optimal preservation of thyroid function (2).

However, the complex cervical anatomy poses inherent risks, particularly potential injury to the cervical sympathetic chain (CSC) during ablation procedures, which may lead to Horner Syndrome (HS) (3). Current major clinical guidelines and consensus statements on ablation therapy make no mention of this complication (2, 4, 5), though the Society of Interventional Radiology categorizes HS as a severe complication in their clinical practice guidelines (6). While the reported incidence of post-ablation HS remains low (fewer than 20 documented cases to date), its occurrence can significantly impact patients’ quality of life and psychological well-being. This review, for the first time, comprehensively integrates existing evidence to elucidate the incidence rate, pathomechanisms, risk factors, and preventive strategies for HS, thereby enhancing the safety profile of ultrasound-guided ablation therapy. Furthermore, it explores future research directions to optimize clinical implementation of this technology.

## 2 Materials and methods

Given limitations in the quantity and quality of available literature, methodological heterogeneity, and challenges in data extraction, a systematic review or meta-analysis was deemed unfeasible. Consequently, this study adopted the PRISMA extension for Scoping Reviews (PRISMA-ScR) as the methodological framework (7). Through comprehensive literature review and structured group discussions, the following key research questions were formulated: (1) Incidence rates of Horner Syndrome (HS) associated with different ablation techniques. (2) Pathomechanisms underlying HS development post-ablation. (3) Identifiable risk factors contributing to HS occurrence. (4) Evidence-based strategies for HS prevention.

### 2.1 Literature search strategy

A comprehensive computerized search was conducted across multiple databases, including Web of Science, PubMed, Cochrane Library, and Embase, covering records from their inception to December 31, 2024. The search strategy incorporated a combination of controlled vocabulary (e.g., MeSH terms) and free-text terms to maximize sensitivity. Key search terms included: (thyroid) AND (Horner Syndrome) AND (ablation therapy OR microwave ablation OR radiofrequency ablation OR high-intensity focused ultrasound OR laser ablation OR percutaneous ethanol injection OR percutaneous lauromacrogol injection). To ensure exhaustive coverage, a snowballing method was implemented by manually reviewing reference lists of included studies to identify additional relevant publications.

### 2.2 Inclusion and exclusion criteria

Inclusion Criteria: (1) Studies reporting ultrasound-guided thyroid ablation therapy using any of the following techniques: MWA, RFA, HIFU, LA, PEI or PLI. (2) Documented occurrence of HS as a post-procedural complication.

Exclusion Criteria: (1) Non-primary research literature, including dissertations/theses, conference abstracts, review articles, or meta-analyses. (2) Cases with pre-existing HS prior to ablation therapy. (3) Studies failing to report HS-related outcomes. (4) Animal studies or *in vitro* experiments. (5) Duplicate publications or studies with inaccessible full texts.

### 2.3 Article selection process

**Figure 1:**
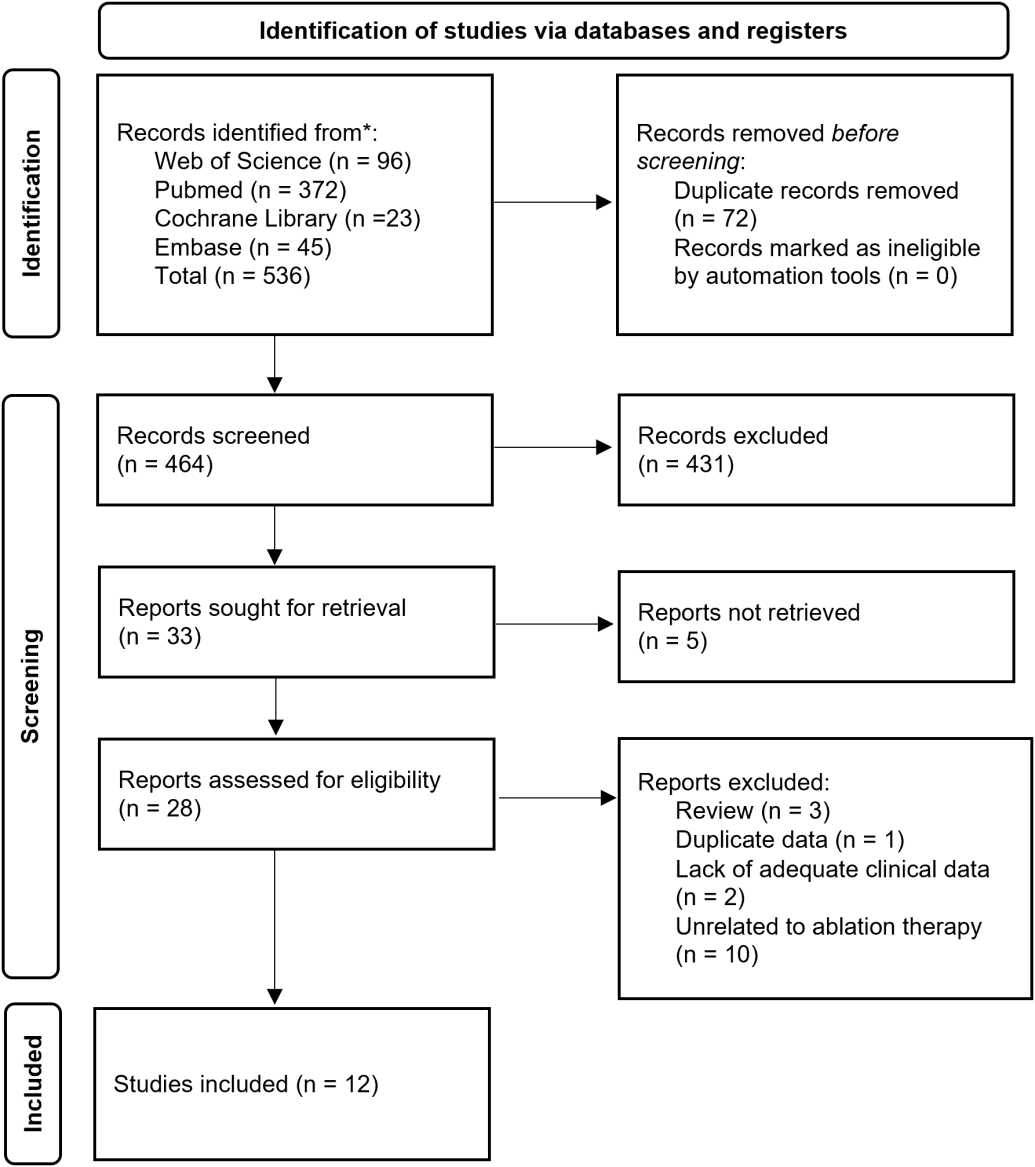
Preferred Reporting Items for Scoping Reviews diagram resembling electronic database search and inclusion/exclusion process of the review. *Date of last search December 31, 2024.

The study selection process was independently conducted by two researchers. First, retrieved citations were organized and duplicates were removed using EndNote software (Clarivate Analytics). Initial screening was performed by reviewing titles and abstracts against predefined inclusion and exclusion criteria. Subsequently, full-text articles of potentially eligible studies were assessed for final eligibility. Following independent evaluations, the researchers cross-checked their selection results. Any discrepancies were resolved through consultation with a senior investigator to achieve consensus. A detailed flowchart of the selection process is provided in **Figure 1**.

## 3 Results

A total of 12 studies (8–19) were included, comprising 3 prospective studies (8–10), 5 retrospective studies (11–15), and 4 case reports (16–19), collectively documenting 13 cases of HS following ultrasound-guided thyroid ablation. All studies were published in English. The distribution of ablation techniques was as follows: MWA accounted for 4 studies (8, 12, 13, 18), RFA for 3 studies (10, 11, 15), HIFU for 3 studies (9, 14, 19), and PEI for 2 studies (16, 17). Notably, no cases associated with LA or PLI were identified. The baseline characteristics of the included studies, including patient demographics, ablation technology, and clinical outcomes, are comprehensively summarized in **Table 1**. The methodological quality of the included studies was evaluated using the JBI Scoping Review Appraisal Tool (20). The results indicated that five retrospective studies carried a risk of selection bias (e.g., non-random grouping), three prospective studies might have compromised statistical power due to small sample sizes, and case reports were constrained by single-center designs.

**Table 1:** The baseline characteristics of the included studies.

Author (year)	Country	Research type	Technology	Sample size	HS cases	Incidence rate (%)	Nature	Follow-up	Outcome
Heck K (2015) (8)	Germany	Prospective follow-up study	WMA	30	1	3.3	Benign	NM	Resolved
Lang BH (2017) (9)	China	Prospective follow-up study	HIFU	30	2	6.7	Benign	4 weeks, 2 months	Resolved
Chuanke S (2024) (10)	China	Prospective follow-up study	RFA	970	1	0.1	NM	NM	NM
Kim C (2017) (11)	Korea	Retrospective cohort study	RFA	875	1	0.1	Benign	6 months	Incomplete resolved
Wu W (2017) (12)	China	Retrospective cohort study	WMA	75	1	1.3	Benign	2 months	Resolved
Vorländer C (2018) (13)	Germany	Retrospective cohort study	WMA	24	1	4.2	Benign	3 months	Resolved
Monpeyssen H(2020) (14)	France, Denmark, Italy	Retrospective follow-up study	HIFU	65	1	1.5	Benign	6 months	Resolved
LaForteza A (2024) (15)	USA	Retrospective follow-up study	RFA	68	1	1.5	Metastatic lymph node	1 month	Resolved
Pishdad GR (2011) (16)	Iran	Case Report	PEI	NM	1	NM	Benign	2 months	Resolved
Ahmed S H (2017) (17)	USA	Case Report	PEI	NM	1	NM	malignant	6 months	Incomplete resolved
Zhang X (2018) (18)	China	Case Report	WMA	250	1	0.4	Benign	5 months	Incomplete resolved
Ben Hamou A (2021) (19)	France	Case Report	HIFU	NM	1	NM	Benign	6 months	Resolved

MWA, Microwave Ablation; HIFU, High-intensity Focused Ultrasound; RFA, Radiofrequency Ablation; PEI, Percutaneous Ethanol Injection; NM, Not mention.

### 3.1 MWA

The four studies investigating MWA-related HS reported the following outcomes: (1) A prospective follow-up study (8) conducted at a single center evaluated MWA for benign thyroid nodules. Among 30 treated patients, 1 case of HS (incidence rate: 3.3%) was observed, with symptoms resolving completely during follow-up. (2) A retrospective case-control study (12) involving 75 patients undergoing MWA for benign thyroid nodules identified 1 HS case (incidence: 1.3%), with full symptom resolution occurring within 2 months. (3) A retrospective cohort study (13) comparing MWA and radiofrequency ablation (RFA) for benign thyroid nodules reported 1 HS case among 24 MWA-treated patients (incidence: 4.2%), demonstrating complete symptom resolution by 3 months post-procedure. (4) A case report (18) documented 1 HS occurrence (incidence: 0.4%) in a cohort of 75 MWA-treated patients with benign nodules, noting partial symptom alleviation at 5-month follow-up.

### 3.2 RFA

The three studies examining RFA-related HS reported the following findings: (1) A prospective follow-up study (10) involving 970 patients undergoing RFA for thyroid nodules documented 1 HS case (incidence: 0.1%), though recovery status was not specified. (2) A retrospective cohort study (11) comparing RFA outcomes in benign thyroid nodules versus recurrent thyroid carcinoma (total cohort: 875 patients) identified 1 HS case (incidence: 0.1%), with partial symptom resolution observed at 6-month follow-up. (3) A retrospective follow-up study (15) evaluating 68 patients receiving RFA for metastatic lymph nodes in thyroid cancer reported 1 HS occurrence (incidence: 1.5%), demonstrating complete symptom resolution within 1 month post-procedure.

### 3.3 HIFU

The three studies investigating HIFU-related HS reported the following outcomes: (1) A prospective follow-up study (9) conducted at a single center evaluated HIFU for refractory Graves’ disease. Among 30 treated patients, 2 cases of HS (incidence: 6.7%) were observed, with complete symptom resolution occurring at 4 weeks and 2 months post-procedure, respectively. (2) A multicenter retrospective follow-up study (14) assessing HIFU for benign thyroid nodules identified 1 HS case (incidence: 1.5%) in a cohort of 65 patients, demonstrating full symptom resolution by 6-month follow-up. (3) A case report (19) documented HS development in a patient undergoing HIFU for a benign thyroid nodule, with complete symptom resolution achieved 6 months post-treatment.

### 3.4 PEI

The two case reports detailing PEI-associated HS are summarized as follows: (1) A case report (16) documented HS in a patient following PEI treatment for metastatic papillary thyroid carcinoma, with partial symptom resolution observed during follow-up. (2) Another case report (17) described HS development after PEI administration targeting level III metastatic lymph nodes, demonstrating partial symptom resolution at 6-month follow-up.

## 4 Discussion

HS, first described in 1869 by Swiss ophthalmologist Johann Friedrich Horner (21), is a clinical triad caused by disruption of the oculosympathetic pathway (OSP). The classic triad comprises ipsilateral ptosis, miosis, and facial anhidrosis. The OSP originates in the hypothalamus and traverses a three-neuron chain to innervate ocular structures, regulating pupillary dilation, eyelid elevation, and ocular motility (3). Of particular relevance to iatrogenic injury is the second-order neuron of this pathway, which constitutes the CSC (3).

Anatomically, the CSC lies superficial to the longus colli muscles, lateral to the vertebral bodies, and deep to the prevertebral fascia. It comprises the superior, middle, and inferior cervical sympathetic ganglia interconnected by nerve fibers. Direct injury to the CSC during thyroid surgery has been documented as a cause of HS (22). While ultrasound-guided ablation therapy demonstrates significant promise for thyroid nodule management, its invasive nature necessitates careful consideration of complications. Although rare, HS has emerged as a clinically significant complication warranting heightened vigilance in interventional practice.

### 4.1 Incidence of HS

As of the search cutoff date, only 13 cases of HS following ablation therapy have been reported. The incidence rates ranged from 0.4% to 4.2% for MWA, 0.1% to 1.5% for RFA, and 1.5% to 6.67% for HIFU. The two case reports on PEI did not specify cohort sizes, precluding calculation of HS incidence for this modality. However, a retrospective cohort study (full text unavailable) (23) mentioned 1 HS case among 250 PEI-treated patients (incidence: 0.4%). Due to the limited number of reported cases and the absence of high-quality randomized controlled trial, current estimates of HS incidence may be subject to inaccuracies or selection bias. Future prospective, large-scale studies are required to establish more precise epidemiological profiles of this complication.

### 4.2 Pathogenetic mechanisms

Thermal ablation techniques share a common mechanism of inducing tumor cell death through protein denaturation and coagulative necrosis via hyperthermia (24). Elevating tissue temperature to 41°C may increase blood flow and enhance ion diffusion across cell membranes (25). Within the hyperthermic range (41°C–48°C), cellular sensitivity to thermal injury intensifies (26), leading to protein unfolding/aggregation and impaired DNA damage repair (27). Irreversible cellular damage at these temperatures typically requires 30 minutes to 1 hour of exposure (28). At higher temperatures (48–60°C), severe protein denaturation occurs (25), with irreversible thermal injury achievable within seconds. Temperatures exceeding 60°C cause near-instantaneous protein denaturation and coagulative necrosis (28). Thermal energy propagation to the CSC during ablation may induce such neurothermal damage (8–12, 14, 18).

Ethanol exerts its ablative effects through tissue dehydration, protein degradation, and thromboembolic effects, which may inadvertently damage adjacent neural structures (29).

Secondary mechanisms include: (1) Mechanical injury: Post-procedural edema or inflammatory responses may compress or traction the CSC, impairing neural conduction (8, 18). (2) Vascular compromise: Ablation-induced vascular injury may disrupt CSC blood supply, resulting in ischemic neuropathy and functional loss (18, 19).

### 4.3 Risk factors

Ablation of nodules or lesions adjacent to the CSC, particularly the MCG, warrants heightened caution (9, 12, 14, 19). The MCG, the smallest ganglion within the CSC, exhibits an ultrasound visualization rate of approximately 50.4%. Anatomically, it is typically located posterior to the carotid sheath and anterior to the longus colli muscles at the C3–C7 vertebral levels, with mean dimensions of 3.8–6.3 mm in width, 1.7–2.1 mm in height, and 6.3 ± 10.5 mm in length (30). HS risk escalates when ablation probe tip approaches the MCG (8, 14) or when the MCG is erroneously targeted during ablation (11). Additionally, large tumor volumes, particularly benign nodules encasing the carotid sheath, increase susceptibility to MCG injury (11). Notably, the MCG resides in proximity to the inferior thyroid artery, whose branches may supply blood to the ganglion (31). Consequently, ablation near the inferior thyroid artery risks compromising the MCG’s vascular supply, potentially inducing neural ischemia.

Carlander et al. (32) demonstrated in a rat model that localized energy delivery from ultrasound devices can induce neural dysfunction, with the extent of nerve injury dependent on thermal exposure duration. Consequently, prolonged ablation duration, higher power settings, or excessive ablation at a single site may amplify thermal accumulation within the ablation zone, thereby increasing the risk of collateral thermal damage to adjacent tissues (13, 14, 19). Tumor tissues, characterized by lower thermal conductivity and impaired heat dissipation, are prone to localized hyperthermia during ablation, leading to necrotic cell death due to their heightened thermosensitivity. In contrast, normal tissues exhibit superior heat dispersion capabilities owing to higher thermal conductivity (24). When the ablation probe tip extends beyond the thyroid nodule margin into surrounding soft tissues, rapid thermal conduction to adjacent structures may occur, potentially damaging the CSC or MCG (8, 13).

The heat sink effect—the phenomenon whereby heat is absorbed and dissipated by highly conductive structures (e.g., blood vessels, airways)—varies across ablation modalities. MWA exhibits less susceptibility to this effect compared to RFA (33), resulting in a more confined thermal field and consequently higher risks to periprocedural tissues in RFA (13). The differences in the risk of inducing HS among different ablation techniques may be attributed to their thermal field distribution characteristics, and further verification is needed regarding the impact of heat conduction patterns on neural injury.

During PEI, ethanol may diffuse into cervical tissues via the needle tract, exerting cytotoxic effects that can induce HS, recurrent laryngeal nerve palsy, and cervical pain (34). In one case report (16), post-PEI cervical ecchymosis—a direct indicator of ethanol extravasation—provided clinical evidence supporting the hypothesis that ethanol leakage contributes to neural injury. The incidence of HS following PEI cannot be accurately estimated due to the lack of large-sample studies. Existing cases suggest that ethanol extravasation may trigger HS through neurotoxic effects, and further research is needed to investigate its dose-effect relationship.

Furthermore, while swallowing facilitates intraprocedural nodule visualization, involuntary swallowing or neck movement during ablation may displace the ablation probe tip, compromising procedural accuracy and safety (19). Anatomical variations also pose risks: communicating branches between the recurrent laryngeal nerve and sympathetic trunk exist in some individuals, and injury to these anastomotic fibers or the recurrent laryngeal nerve itself may disrupt CSC conduction (12, 35).

### 4.4 Preventive strategies

For thermal ablation procedures, meticulous selection of device parameters and treatment zones is critical to avoid complications such as HS (14). Preprocedural evaluation should include detailed assessment of the spatial relationship between thyroid nodules and the MCG (8, 9, 13, 19). Real-time ultrasound-guided localization of the MCG and preoperative mapping of the CSC trajectory are essential to minimize intraprocedural neural injury (8, 11, 13).

Technical parameters and ablation strategies should be optimized based on nodule size and location to mitigate risks to adjacent critical structures (11, 13). The “moving shot technique”—a dynamic ablation approach involving sequential probe repositioning—can prevent excessive thermal accumulation at a single site (12, 13). For nodules in proximity to neural structures, incomplete ablation may be considered, prioritizing neural preservation over maximal volume reduction (8, 12).

The creation of a hydrodissection-induced fluid barrier between the tumor and critical neural structures (8, 12, 13, 19) can mitigate thermal injury risks by maintaining perineural temperatures below 45°C (distinct from the ablation zone temperature) (36). Rational application of this isolation technique not only prevents unintended thermal diffusion and minimizes postprocedural adhesion risks but also expands interfascial spaces to enhance procedural maneuverability (37).

During ablation, precise probe positioning must ensure the ablation probe tip remains entirely within the nodule, avoiding protrusion beyond the lesion margins (11, 13). Advanced probe designs incorporating distal choke collars or integrated cooling systems may further reduce thermal dispersion and heat sink effects, thereby lowering complication rates (38).

Continuous intraprocedural monitoring for early signs of HS, particularly ocular or conjunctival discomfort/pain, is critical. Such symptoms may serve as early predictive indicators, facilitating real-time identification of “danger zones,” especially those adjacent to the CSC (19). Employing general anesthesia (rather than local anesthesia) enables intraoperative assessment of eyelid ptosis—a key diagnostic marker of HS—allowing immediate procedural adjustments (18).

Preprocedural patient education must emphasize detailed disclosure of potential complications, including HS, with explicit documentation of informed consent obtained prior to intervention (11, 13).

### 4.5 Future directions

Based on the existing evidence, future research should focus on the following directions: (1) Establishing a risk prediction model for HS, integrating ultrasonic anatomical parameters with ablation energy settings; (2) Evaluating the neuroprotective effects of novel cooled probes; (3) Conduct long-term follow-up to clarify the impact of HS on the ablation population.

Emerging technologies hold transformative potential for optimizing ablation safety and efficacy. Augmented reality systems, which convert 2D imaging into 3D holographic reconstructions, may enable precise preoperative mapping of thyroid anatomy (including tumors, vasculature, and neural structures) through the integration of artificial intelligence-driven segmentation and 3D-printed anatomical models, thereby enhancing preprocedural planning and real-time intraoperative navigation (39–41).

Further innovation lies in combining fluid barrier techniques with contrast-enhanced lymphography to dynamically track lymphatic drainage patterns. This hybrid approach could improve metastatic lymph node detection accuracy and facilitate the development of standardized protocols for pre-ablation assessment and management of cervical lymph node metastases (42).

Blood perfusion rate significantly influences ablation efficacy. Systematic investigation of perfusion heterogeneity across thyroid nodule subtypes could enable personalized parameter optimization (e.g., power output, duration) to achieve optimal therapeutic outcomes (43). Development of predictive models for estimating post-ablation volume reduction ratios would support clinicians in formulating tailored ablation strategies and providing evidence-based prognostic counseling to patients (44, 45).

Advanced multi-layer thermal monitoring systems capable of real-time temperature mapping across tissue layers, anatomical regions, and adjacent organs are urgently needed. Concurrent innovation in thermoprotective materials and precision insulation technologies may further enhance procedural safety by containing thermal spread within predefined ablation zones (43).

## 5 Conclusions

Although HS following thyroid ablation exhibits a relatively low incidence rate, its potential impact on patient quality of life necessitates heightened clinical vigilance. The risk of HS can be effectively mitigated through optimized ablation techniques, enhanced preoperative anatomical mapping, and real-time intraoperative monitoring of critical neural structures. These refinements further improve the safety and efficacy profiles of ultrasound-guided percutaneous ablation therapy for thyroid nodules. Future high-quality prospective studies are imperative to establish precise epidemiological data on HS incidence, characterize long-term neurological sequelae, and validate risk stratification protocols. Such evidence will strengthen evidence-based clinical guidelines and advance personalized therapeutic strategies in interventional thyroidology.
